# An Evil Backstage Manipulator: Psychological Factors Correlated with Health-Related Quality of Life in Chinese Patients with Crohn's Disease

**DOI:** 10.1155/2013/464698

**Published:** 2013-12-26

**Authors:** Song Liu, Jianan Ren, Zhiwu Hong, Xiaoting Li, Min Yao, Dongsheng Yan, Huajian Ren, Xiuwen Wu, Gefei Wang, Guosheng Gu, Qiuyuan Xia, Gang Han, Jieshou Li

**Affiliations:** ^1^Department of Surgery, Jinling Hospital, Medical School of Nanjing University, Nanjing 210002, China; ^2^Center for the Study of Inflammatory Bowel Disease, GI Unit, Department of Medicine, Massachusetts General Hospital and Harvard Medical School, Boston, MA 02114, USA; ^3^Department of Surgery, Jinling Hospital, Nursing College, Nanjing University of Chinese Medicine, Nanjing 210002, China; ^4^Department of Surgery, Limb Preservation and Wound Care Research, VA New England Health Care Division, Providence, RI 02908, USA; ^5^Department of Pathology, Jinling Hospital, Medical School of Nanjing University, Nanjing 210002, China; ^6^Department of General Surgery, Second Affiliated Hospital of Jilin University, General Surgery Center of Jilin University, Changchun 130000, China

## Abstract

Health-related quality of life (HRQoL) is recommended as one of essential parameters to evaluate treatment effect and clinical outcome in patients with Crohn's disease (CD). Recent studies reported that psychological factors might play a role in HRQoL in Western and American CD patients. Sufficient evidences in Chinese CD patients are still unavailable. This study is dedicated to investigate the correlation of various psychological factors with HRQoL in Chinese CD patients. We prospectively collected 40 active and 40 quiescent CD patients in China and found that psychological factors, especially neuroticism and anxiety, significantly correlate with and affect HRQoL in both active and quiescent CD groups. This is the first report revealing correlation between psychological factors and HRQoL in Chinese CD patients. Therefore, we assume that our results can contribute to a better understanding of etiology and tailoring of management in Chinese patients with Crohn's disease and are beneficial to our colleagues to compare the heterogeneous characteristics of Crohn's disease in different ethnic groups.

## 1. Introduction

Crohn's disease (CD) is characterized by chronic, transmural intestinal inflammation in which periods of remission with variable length are interrupted by relapse episodes [1]. Health-related quality of life (HRQoL) has been broadly defined as a concept that includes emotional, social, and physical dimensions of health functioning [2]. Improvement in quality of life in patients with CD has been increasingly attracting attention of clinical physicians. Inflammatory Bowel Disease Questionnaire (IBDQ) was therefore developed and being widely used to comprehensively assess healthy status and HRQoL in patients with inflammatory bowel disease (IBD) [3, 4]. On the basis of the assumption that IBDQ could effectively reflect patient's symptom load, psychological fluctuation, and quality of life [4], IBDQ has also been recommended as one of essential instruments to evaluate treatment effect and clinical outcome [5].

In recent decades, a series of studies performed in Europe and North America reported several distinct psychological traits that relate to HRQoL and outcome of surgery in patients with CD [2, 4, 6, 7]. These findings intensively suggest that some disease-unrelated variables may affect IBDQ score as well and, consequently, it is necessary to consider these variables into the evaluation of HRQoL and figure out to what extent that these variables would influence clinical outcome in patients with CD.

To our knowledge, there is no report investigating correlation between psychological factors and HRQoL in Chinese patients with CD. Considering the heterogeneous characteristics of Crohn's disease in different ethnic groups reported before, we assumed that there is a necessity to perform an investigation in Chinese population. Therefore, current study was dedicated to analyze the correlation between various psychological factors and HRQoL in patients with CD from China. All questionnaires adopted in current study have been validated for Chinese population.

## 2. Methods

### 2.1. Patients

Patients in current study were recruited from outpatient clinic of CD in Jinling Hospital between March 2012 and August 2012. Patients were first screened by a physician for eligibility assessment. Then, their blood sample would be collected for C-reactive protein (CRP), erythrocyte sedimentation rate (ESR), and whole blood analysis. Afterwards, they would be evaluated by another physician for disease activity using CDAI score. Subsequently, patients were required to accomplish all questionnaires (self-reporting) truthfully and independently, and then they were checked by a clinical nurse.

All enrolled patients were 18–65 years old and diagnosed as CD by clinical manifestations, radiologic, endoscopic, and histopathologic evidences. The exclusion criteria included current or previous mental disorders, receiving or used to receive psychotherapy, presence of major medical conditions or chronic disease history other than inflammatory bowel disease, and unwilling to participate in or cannot accomplish current study.

### 2.2. Health-Related Quality of Life and Psychological Evaluation

IBDQ is the most widely used and validated disease-specific instrument [3], which contains 32 items and assesses four aspects (subscales) of health-related quality of life: emotional function (12 items), social function (5 items), bowel function (10 items), and systemic symptom (5 items) [8]. Each item is scored with 7-level Likert scale; the higher score, the better health-related quality of life [4]. This questionnaire could provide a total score and four subscale scores of each aspect. The IBDQ used in current study has been translated and validated in Chinese population [9].

To assess neuroticism and social conformity and desirability, the Neuroticism and Lie subscales from the Eysenck Personality Inventory (EPI) were adopt in current study [10]. Twenty-three items are designed to evaluate neuroticism (Neuroticism subscale) and the other 21 items are assigned for social desirability (Lie subscale). The reliability and validity of this scale are well established in Chinese population [11].

The Hospital Anxiety and Depression Scale (HADS) was first developed in 1983 [12] and has been extensively used in subsequent studies to identify the potential anxiety and depression in nonpsychiatric hospitalized patients [13]. This questionnaire includes separated Anxiety and Depression subscales with seven items, respectively. Previous studies have confirmed the validity of this scale among Chinese patients [14, 15].

To determine the dynamics of aggression among individuals, the Buss-Perry Aggregation (BPA) questionnaire was employed in this study [16]. BPA has been one of the most popular self-report inventories for the measurement of four different dimensions (anger, hostility, physical aggression, and verbal aggression) during the past two decades since its publication in 1992 [17]. This 29-item questionnaire is scored with a 5-level scale for each question, contributing to a total score and four subscales of each dimension. A higher score suggests a higher level of aggression feelings [4]. BPA has been validated in Chinese version as well [17].

### 2.3. Statistical Consideration

All statistical tests were performed with SPSS software (SPSS for Windows, version 13.0, SPSS, Chicago, IL). All analyses were 2-tailed and differences were considered statistically significant when *P* values <0.05. Continuous variables were described as mean ± SD and compared with unpaired student's *t*-test. Categorical variables were presented as percentages and compared with Fisher's exact test or Chi-square test. Hierarchical multiple regression was performed to analyze the effect of different psychological factors on HRQoL independent of the influence of other parameters, such as disease activity (CDAI score). In this model, dependent parameters were IBDQ and its subscales; independent factors included demographic parameters (including age, gender, years of education, and duration of disease) entered in step 1, and CDAI score entered in step 2 as a control parameter, and then all psychological factors entered in step 3. Spearman correlation analysis was performed to investigate the correlation between psychological parameters and IBDQ and its subscales. Multiple linear regression analysis with forward stepwise methods was used to investigate to what extent that psychological factors could affect IBDQ and its subscales. Psychological variables that displayed a significant correlation with IBDQ or its subscales were entered in the regression analysis.

### 2.4. Ethical Statement

This study was approved by the Medical Ethics Committee of Jinling Hospital. A written informed consent was obtained from each enrolled patient.

## 3. Results

**Figure 1:**
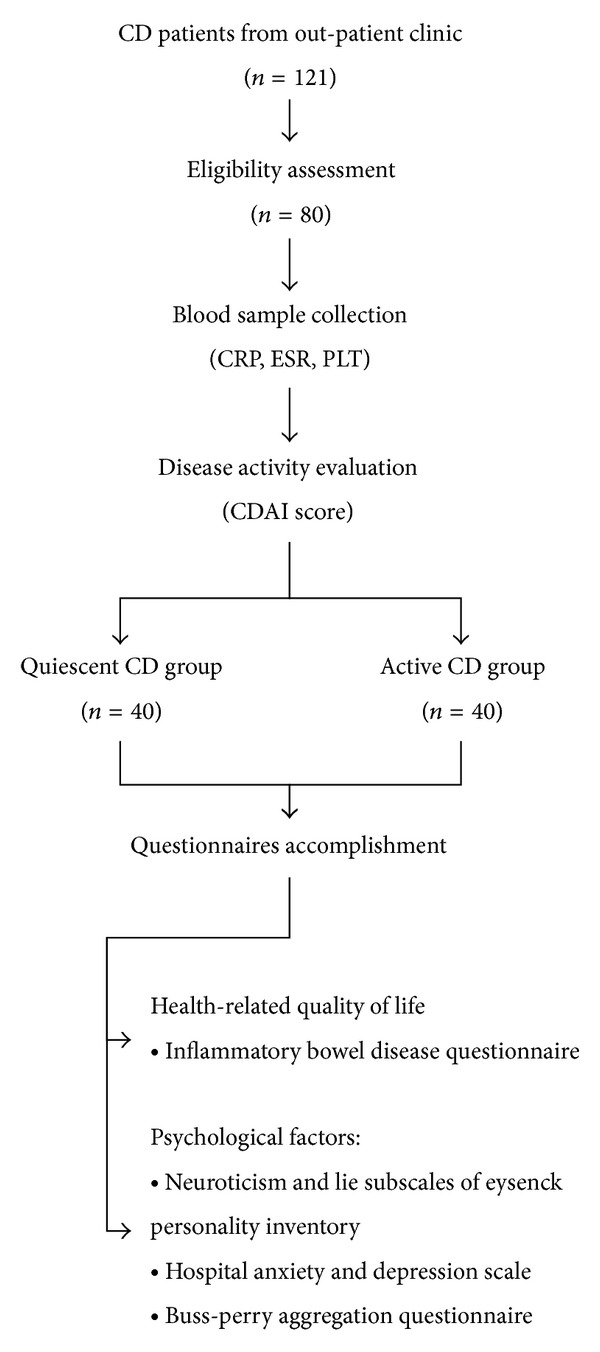
Study design of current prospective study. A total of 121 patients with Crohn's disease from outpatient clinic were recruited in the current study. After eligibility assessment, 80 eligible patients were enrolled. Their blood samples were collected for CRP, ESR, and PLT analysis. Afterwards, they were evaluated for disease activity with Crohn's disease activity index (CDAI score) and then divided into quiescent (CDAI ≤ 150 points) and active (CDAI > 150 points) CD groups. All enrolled patients were subsequently required to accomplish HRQoL-related questionnaire (Inflammatory Bowel Disease Questionnaire, IBDQ) and psychological factor-related questionnaires, including Neuroticism and Lie subscale from Eysenck Personality Inventory (EPI), Hospital Anxiety and Depression Scale (HADS), and Buss-Perry Aggregation (BPA) questionnaire. CD: Crohn's disease.

A total of 121 patients diagnosed with CD visited our clinics during the period of study. According to the exclusion criteria, 80 patients were eligible and finally enrolled (Figure 1).

To better investigate the correlation between psychological factors and HRQoL in these patients, we firstly performed a hierarchical multiple regression analysis to discover and then exclude potential confounding parameters. According to Table 1, demographics (step 1) did not contribute to IBDQ or any of its subscales. However, when CDAI score was entered into this model (step 2), *R* square elevated dramatically to a statistical significant degree, indicating that disease activity was a significant covariant in the relationship between psychological factors and HRQoL. Notably, when psychological factors were entered into this model (step 3), the *R* square increased again, demonstrating that a predictive power was added to the model by the addition of psychological factors (Table 1).

Therefore, to reduce the confounding effect of disease activity in the relationship between psychological factors and HRQoL [18–20], we established two independent groups with different disease activity (active CD and quiescent CD), measured by Crohn's disease activity index (CDAI), and analyzed them separately. Among them, 40 patients were assigned into active CD group (CDAI ≤ 150 points) and the other 40 participates were in quiescent CD group (CDAI > 150 points). Both of them displayed a male predominance, which was in accordance to our previous investigations [21]. The CDAI scores were 91.8 points and 247.8 points, respectively, suggesting a distinct variance of disease activity between the two groups (*P* < 0.001). Meanwhile, CRP (*P* < 0.001) and ESR (*P* = 0.022) levels increased significantly in active group while platelet count was similar between these groups.  Table 2 described demographics and clinical characteristics of all enrolled subjects.

The mean duration of CD was 2.90 and 4.36 years for each group. The majority of enrolled patients in both groups had never received operation or less than 1 operation. 5-aminosalicylic acid (5-ASA), that mainly includes sulfasalazine and mesalazine, was prescribed to the majority of patients in both groups. TWP (*Tripterygium wilfordii* polyglycoside) is a traditional Chinese medicine which has been confirmed to be effective in the management of CD [22, 23] and therefore becomes a choice of medication prescriptions in our institute.

Table 3 demonstrated all psychological scores and IBDQ score and its subscales in both groups. Patients with active CD displayed a significant higher level of neuroticism, anxiety, depression, physical aggression, and hostility, paralleled to a significant decrease in HRQoL when compared to patients in quiescent CD group (Table 3). Meanwhile, there was not any statistical difference between groups in social desirability and anger (Table 3). Interestingly, verbal aggression seems to be more apparent in patients with quiescent CD (Table 3).

We further examined the correlation between various psychological factors and IBDQ together with its subscales in separate analyses of active CD (Table 4) and quiescent CD (Table 5) groups.

In the former group, neuroticism, anxiety, and depression were negatively correlated with IBDQ and its emotional function, bowel function, and systemic symptom subscales. Simultaneously, BPA was correlated with IBDQ and its emotional subscale, whilst physical aggression was correlated with IBDQ bowel function; anger and hostility were confirmed to correlate with IBDQ emotional subscale. However, social desirability did not correlate with any HRQoL parameters, while IBDQ social function did not correlate with any of the psychological variables (Table 4).

In the latter group, neuroticism, anxiety, and depression were significantly correlated with IBDQ and its all four subscales. Moreover, hostility was found to correlate with IBDQ and its emotional and social function subscales. There was not any other correlations in these patients (Table 5).

We subsequently employed multiple linear regression analysis to investigate to what extent that psychological factors could affect IBDQ and its each subscale. Psychological factors that displayed a statistical correlation with IBDQ or any subscale in previous correlation analysis were introduced as independent parameters into this regression model.  Table 6 illustrated the results of regression analysis.

In quiescent CD group, a higher level of neuroticism leads to lower scores of IBDQ, IBDQ emotional function, and IBDQ bowel function. Meanwhile, a higher level of anxiety leads to a lower score of IBDQ systemic symptom (Table 6).

In active CD group, neuroticism was similarly confirmed as a remarkable factor to negatively affect IBDQ, IBDQ social function, IBDQ bowel function, and IBDQ systemic symptom. Moreover, IBDQ emotional function in this group was found to simultaneously interact with both neuroticism and anxiety. All interactions in this regression analysis were negative (Table 6).

## 4. Discussions

To the best of our knowledge, this is the first report investigating correlation between psychological factors and HRQoL in Chinese patients with Crohn's disease. Similar studies have been performed in Europe and North America. Considering the heterogeneous characteristics of Crohn's disease in different ethnic groups reported before, we believe that our description of psychological traits and their correlation with HRQoL in Chinese patients would be beneficial for our colleagues and would contribute to a better understanding of etiology and tailoring of management in Chinese patients with Crohn's disease.

In current study, we discovered that disease activity could exert significant influence on HRQoL in the presence of psychological factors. This was in accordance with previous studies [24]. Vidal and his colleagues [25] confirmed disease activity as one of strongest predictors of HRQoL impairment. Haapamaki [26] discovered disease activity as a significant factor related to HRQoL impairment. Other studies also reported an adverse effect of disease activity on HRQoL in CD patients [27].

After dividing enrolled patients into two independent groups, we discovered that active CD patients were exposed to significant higher risks of neuroticism, anxiety, depression, physical aggression, and hostility, together with a significant decrease in HRQoL compared to patients in quiescent CD. In correlation analysis, we confirmed that psychological factors, particularly neuroticism, anxiety, and depression, are related to IBDQ and its subscales in both active and quiescent groups.

Neuroticism was demonstrated as a significant variable that leads to declined scores of IBDQ, IBDQ emotional function, and IBDQ bowel function in quiescent CD group and IBDQ, IBDQ social function, IBDQ bowel function, and IBDQ systemic symptom in active group. This is in line with the findings from previous studies of IBD [4, 6] and other disorders [28–30]. We believe that neuroticism should result from both genetic factor and somatic conditions and could contribute to vulnerable reaction with depressive symptoms to stressful life events in these patients [4, 31], which may be helpful to explain the correlation between neuroticism and IBDQ emotional function [32].

Several previous studies have investigated the association between neuroticism and IBDQ and its subscales [4, 33]. They consistently confirmed the remarkable role of neuroticism in the influence of HRQoL on IBD patients. However, they perform analyses in mixed IBD samples and not effectively excluded the potential effect that disease activity exerted on HRQoL. In our study, we divided patients into quiescent and active CD groups and analyzed them separately to reduce the interactions among disease activity, psychological factors, and HRQoL in these patients. In fact, our results confirmed the distinct variance of psychological scores between active and quiescent CD groups (Table 2), making it reasonable to perform separate analyses which could decrease the bias brought by mixed samples of patients with various disease activities.

Our study found anxiety to be a significant factor that leads to declined scores of IBDQ systemic symptom in quiescent CD and IBDQ emotional function (together with neuroticism) in active CD patients. Prior studies demonstrated some controversies regarding the role that anxiety plays in IBD patients. While some studies reported a prevalence of anxiety as high as 29–35% in remission and 80% in relapse [34], others found no evidence of any correlation between anxiety and CD [35]. A recent study in Korea reported an incidence of 27.4% in IBD patients, accompanied with a significant lower quality of life in these patients compared with healthy controls [36]. We agree with the authors that anxiety could originate from various concerns and worries about incurability and uncertain course and prognosis in Crohn's disease, as well as fear of surgery or development of cancer [24, 37].

Our results suggested that in both quiescent and active CD patients, an increased level of neuroticism and anxiety might reduce the total IBDQ and some other subscales at a magnitude that might be of clinical significance. Therefore, psychological factors, especially neuroticism and anxiety, should be taken in account when using IBDQ to measure clinical outcome of interventions in patients with CD.

We confirmed in current study that psychological distress, concerns, and illnesses could lead to decreased HRQoL and should draw attention of professional staff. However, the majority of current strategies for CD did not pay attention to psychological conditions or concerns in CD patients [24]. Instead, some of their side effects were associated with mental or mood changes, depressions, and other psychological distresses. Therefore, it could be beneficial if integrating conventional medical therapy with psychological interventions improves HRQoL in patients with CD.

We are aware of the limitations in this study. First, a potential risk of selection bias might exist because of the limited sample size from a single center, which may not completely reflect the comprehensive relationship between psychological factors and HRQoL.

Second, this study lacks follow-up prospective data after receiving psychological interventions in all enrolled patients, and thus we were deprived of the ability of evaluating efficacy of psychological treatments in CD patients. However, Boye and his colleagues [38] in their INSPIRE study declared that psychotherapy could improve quality of life in UC but not in CD. Therefore, role of psychotherapy needs to be determined by further studies.

Third, a disease-specific HRQoL questionnaire (IBDQ) was adopted in current study, but not a generic questionnaire, such as SF-36. McColl [39] recommended that generic and disease-specific measures of quality of life are complementary and thus should be used in parallel in IBD. We agree with McColl's suggestion, but we tried to reduce the total time that all questionnaires take in each participant, therefore assisting them to answer all questions truthfully and accurately in this self-reporting investigation.

In conclusion, psychological factors, especially neuroticism and anxiety, can significantly affect HRQoL in patients with both active and quiescent CD. Therefore, more attention toward psychological status in Crohn's patients is required in current and future management. Furthermore, continued large studies are expected to enhance our capacity to design interventions to improve HRQoL and maximize health outcomes in the management of Crohn's disease in the future.

## Figures and Tables

**Table 1:** Analysis of potential confounding parameters in the relationship between psychological factors and HRQoL in patients with Crohn's disease.

	Step 1 (demographics*)		Step 2 (CDAI score)		Step 3 (psychological factors^&^)	
	*R* square	*P* value	*R* square	*P* value	*R* square	*P* value
IBDQ	0.060	0.195	0.470	<0.001	0.747	<0.001
IBDQ-E	0.081	0.090	0.376	<0.001	0.780	<0.001
IBDQ-SF	0.018	0.709	0.196	0.002	0.345	0.002
IBDQ-B	0.040	0.367	0.446	<0.001	0.656	<0.001
IBDQ-SS	0.052	0.252	0.531	<0.001	0.701	<0.001

*Demographics included age, gender, years of education, and duration of disease; ^&^psychological factors included Neuroticism and Lie scores, Hospital anxiety and depression scores, and Buss-Perry score and its subscales; IBDQ-E: IBDQ emotional function; IBDQ-SF: IBDQ social function; IBDQ-B: IBDQ bowel function; IBDQ-SS: IBDQ systemic symptom.

**Table 2:** Demographics and clinical characteristics in patients with Crohn's disease.

	Quiescent CD (CDAI* ≤ 150 points)	Active CD (CDAI > 150 points)	*P *
Number of patients	40	40	—
Male (%)	30 (75.0)	31 (77.5)	ns
Age (years)	29.2 ± 10.1	35.2 ± 11.4	0.015
Education (years)	13.9 ± 2.92	12.0 ± 3.55	ns
Smoking history			ns
Never	27	24	
Past smoker	12	11	
Current smoker	1	5	
Duration of disease (years)	2.90 ± 2.99	4.36 ± 4.23	ns
Times of previous gastrointestinal operations			ns
0	20	17	
1	13	14	
2	6	3	
≥3	1	6	
Disease location			ns
L1 (ileal)	18	18	
L2 (colonic)	8	10	
L3 (ileocolonic)	14	12	
+L4 (upper gastrointestinal)	2	2	
Disease behavior			ns
B1 (inflammatory)	10	10	
B2 (stricturing)	24	23	
B3 (penetrating)	6	7	
+P (perianal)	18	17	
Medications^&^			ns
None	5	0	
5-ASA^#^	22	26	
Azathioprine	6	6	
TWP^%^	4	6	
Enteral nutrition	18	17	
CDAI score	91.8 ± 32.3	247.8 ± 80.2	<0.001
C-reactive protein (mg/L)	17.5 ± 5.32	61.01 ± 12.99	<0.001
Erythrocyte sedimentation rate (mm/hour)	22.4 ± 4.40	37.34 ± 5.29	0.022
Platelet count (×10^9^/L)	202 ± 9.88	289 ± 17.5	ns

*Crohn's disease activity score; ^&^sum may be more than 100% as one patient may be prescribed with several medications; ^#^5-aminosalicylic acid mainly includes sulfasalazine and mesalazine; ^%^ *Tripterygium wilfordii* polyglycoside; ns: not significant.

**Table 3:** Psychological variables and HRQoL in patients with Crohn's disease.

	Quiescent CD (CDAI* ≤ 150 points)	Active CD (CDAI > 150 points)	*P *
Neuroticism score	8.08 ± 5.02	11.0 ± 5.83	0.019
Lie (social conformity/desirability) score	13.5 ± 2.73	12.7 ± 3.06	ns
Hospital anxiety score	5.20 ± 2.91	7.30 ± 3.14	0.003
Hospital depression score	5.03 ± 3.17	6.98 ± 3.08	0.007
Buss-Perry score	69.7 ± 14.0	79.1 ± 16.9	0.008
PA (physical aggression) score	19.5 ± 4.79	21.9 ± 5.79	0.049
VA (verbal aggression) score	19.5 ± 5.25	15.6 ± 3.93	0.011
A (anger) score	13.4 ± 3.64	18.7 ± 5.21	ns
H (hostility) score	17.3 ± 6.54	22.9 ± 5.78	0.007
IBDQ^&^	181.4 ± 22.3	146.0 ± 31.9	<0.001
Emotional function score	69.7 ± 9.79	57.5 ± 12.8	<0.001
Social function score	23.0 ± 7.01	18.6 ± 7.05	0.006
Bowel function score	60.5 ± 6.68	49.9 ± 9.53	<0.001
Systemic symptom score	28.2 ± 4.68	20.0 ± 5.73	<0.001

*Crohn's disease activity score; ^&^Inflammatory Bowel Disease Questionnaire; ns: Not significant.

**Table 4:** Spearman correlation analysis (*P* value) between different psychological variables and Inflammatory Bowel Disease Questionnaire and its subscales in patients with quiescent Crohn's disease (CDAI ≤ 150 points).

	Quiescent CD (CDAI ≤ 150 points)				
	IBDQ	IBDQ-E	IBDQ-SF	IBDQ-B	IBDQ-SS
Neuroticism	−0.617***	−0.768***	**−**0.116	−0.389*	−0.476**
Lie (social conformity/desirability)	0.134	0.149	0.038	0.063	0.128
Hospital anxiety	−0.421**	−0.562***	0.157	−0.392*	−0.522***
Hospital depression	−0.469**	−0.537***	0.012	−0.428**	−0.464**
Buss-Perry score	−0.330*	−0.481**	0.056	**−**0.309	**−**0.261
PA (physical aggression)	**−**0.196	**−**0.208	0.077	−0.362*	**−**0.137
VA (verbal aggression)	**−**0.142	**−**0.135	**−**0.051	**−**0.124	**−**0.215
A (anger)	**−**0.249	−0.405**	0.078	**−**0.231	**−**0.168
H (hostility)	**−**0.266	−0.465**	0.059	**−**0.166	**−**0.208

IBDQ-E: IBDQ emotional function; IBDQ-SF: IBDQ social function; IBDQ-B: IBDQ bowel function; IBDQ-SS: IBDQ systemic symptom. *P < 0.05; **P < 0.01; ***P < 0.001. The bold data indicates a statistical significance in the Spearman correlation analysis.

**Table 5:** Spearman correlation analysis (*P* value) between different psychological variables and Inflammatory Bowel Disease Questionnaire and its subscales in patients with active Crohn's disease (CDAI > 150 points).

	Active CD (CDAI > 150 points)				
	IBDQ	IBDQ-E	IBDQ-SF	IBDQ-B	IBDQ-SS
Neuroticism	−0.690***	−0.745***	−0.651***	−0.578***	−0.452**
Lie (social conformity/desirability)	0.198	0.240	0.144	0.193	0.222
Hospital anxiety	−0.572***	−0.669***	−0.543***	−0.370*	−0.394*
Hospital depression	−0.463**	−0.560***	−0.399*	−0.363*	−0.330*
Buss-Perry score	**−**0.251	**−**0.238	**−**0.208	**−**0.163	**−**0.125
PA (physical aggression)	0.002	**−**0.034	**−**0.025	0.002	**−**0.024
VA (verbal aggression)	**−**0.080	**−**0.034	**−**0.076	**−**0.111	0.087
A (anger)	**−**0.289	**−**0.272	**−**0.162	**−**0.281	**−**0.234
H (hostility)	−0.434**	−0.467**	−0.388*	**−**0.226	**−**0.292

IBDQ-E: IBDQ emotional function; IBDQ-SF: IBDQ social function; IBDQ-B: IBDQ bowel function; IBDQ-SS: IBDQ systemic symptom. *P < 0.05; **P < 0.01; ***P < 0.001. The bold data indicates a statistical significance in the Spearman correlation analysis.

**Table 6:** Multiple linear regression analysis between different psychological variables and Inflammatory Bowel Disease Questionnaire and its subscales in patients with Crohn's disease.

	Quiescent CD (CDAI ≤ 150 points)					Active CD (CDAI > 150 points)				
	Significant variables	Adjusted *R* square	*B*	95% CI	*P* value	Significant variables	Adjusted *R* square	*B*	95% CI	*P* value
IBDQ	Neuroticism	0.363	−2.73	−1.59~−3.88	<0.001	Neuroticism	0.496	−3.90	−2.64~−5.16	<0.001
IBDQ-E	Neuroticism	0.548	−1.46	−1.03~−1.88	<0.001	Neuroticism	0.560	−1.10	−0.53~−1.67	<0.001
						Anxiety	0.639	−1.60	−0.54~−2.65	0.004
IBDQ-SF						Neuroticism	0.430	−0.81	−0.51~−1.10	<0.001
IBDQ-B	Neuroticism	0.191	−0.61	−0.22~−1.00	0.003	Neuroticism	0.336	−0.97	−0.54~−1.40	<0.001
IBDQ-SS	Anxiety	0.290	−0.89	−0.45~−1.33	<0.001	Neuroticism	0.194	−0.46	−0.17~−0.74	0.003

IBDQ-E: IBDQ emotional function; IBDQ-SF: IBDQ social function; IBDQ-B: IBDQ bowel function; IBDQ-SS: IBDQ systemic symptom.
